# Mitral regurgitation: when to intervene? A historical perspective

**DOI:** 10.1007/s12471-020-01417-x

**Published:** 2020-04-14

**Authors:** A. Bergstra, C. Simsek, A. F. M. van den Heuvel

**Affiliations:** 1 Department of Cardiology, University Medical Centre Groningen, University of Groningen, Groningen, The Netherlands; 2 Department of Cardiology, Amphia Ziekenhuis, Breda, The Netherlands

**Keywords:** Mitral regurgitation, Haemodynamics, Wall stress, MitraClip procedure

## Abstract

Although mitral regurgitation (MR) is the most common valvular heart disease, it should be regarded as a complex multifactorial disease that involves multiple entities. Optimal medical therapy alone does not hinder the progression of the disease, and in the 1980s it was already recognised that corrective surgery is indicated if MR is severe and patients are symptomatic (except for those with the most severe left ventricle dysfunction). Later on, asymptomatic patients with deterioration of the left ventricular ejection fraction were also operated on to avoid irreversible left ventricular dysfunction, heart failure and eventually death. However, a major drawback remains the fact that a significant group of patients is considered to have a high perioperative risk due to their advanced age or severe comorbidities. Since less invasive, percutaneous interventions have been developed and recently thoroughly investigated in the MITRA-FR and the COAPT studies, the type of intervention and also the timing have become more crucial. In this critical review of the literature, we describe what we should have learned from the past and which (haemodynamic) parameters can best predict the outcome in patients with MR.

## Introduction

Until the 1980s, mitral regurgitation (MR) was mainly diagnosed by right heart catheterisation in combination with left ventricular cine-angiography. Increased right-sided pressures, such as pulmonary artery and pulmonary wedge pressure, and in particular the presence of an early and/or high V wave, suggested the presence of MR. Moreover, an enlarged left ventricle as well as left atrium could be seen during left ventricular (LV) angiography. The grade of MR was mainly estimated from the shape and volume of contrast fluid in the left atrium. All of these values were used as criteria for surgery. The advancement of echocardiographic techniques, such as Doppler measurements, offered additional diagnostic possibilities and eventually largely replaced invasive and angiographic methods in daily clinical practice.

Although considerations were made carefully with the aforementioned entities in the past, the postoperative results were sometimes disappointing. In order to search for criteria that could predict surgical success, several studies on the haemodynamics of MR have been published [1–3]. Current guidelines state that surgical intervention should be based on the severity of MR, patient symptoms, LV systolic function ≤60% or LV end-systolic diameter (LVESD) ≥45 mm, presence of atrial fibrillation and systolic pulmonary artery pressure of more than 50 mm Hg [4]. However, at the same time, patients referred to undergo surgery earlier in the natural process of the disease have been shown to have a better outcome in terms of survival compared to patients that were treated according to the criteria in these guidelines [5]. Nowadays, a trend is observed to advise patients to undergo surgery when there is a high likelihood of successful mitral valve (MV) repair (if LVESD ≥40 mm and flail leaflet and/or left atrial (LA) volume ≥60 ml/m^2^).

Not only are the criteria for MV surgery changing continuously, but the introduction of novel percutaneous edge-to-edge transcatheter MV repair techniques (MitraClip, Abbott Vascular, Menlo Park, CA, USA) also warrants comprehensive knowledge on the part of the treating physicians. Lessons learned from the past in haemodynamics and MV surgery could be essential for further improvements in patient selection and timing of the procedure.

## Haemodynamics in MR

**Fig. 1:**
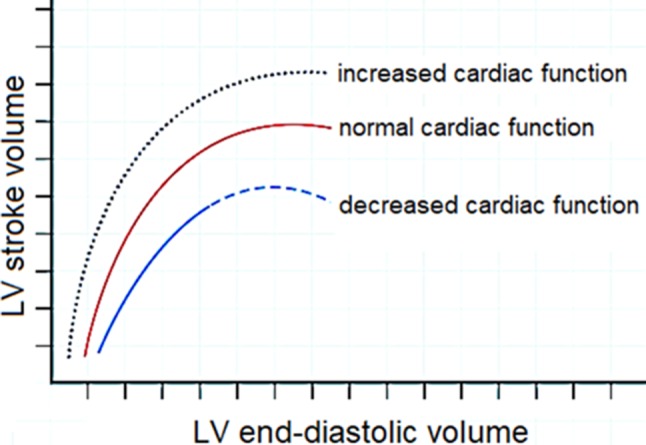
The Frank-Starling relationship is the observation that ventricular output increases as preload (end-diastolic volume) increases. *LV* left ventricular

The Frank-Starling curve shows the relation between LV stroke volume (LVSV) and LV end-diastolic volume (LVEDV) (Fig. 1). The essence of this curve is the fact that the LVSV increases as a response to an increased LVEDV, which is caused by stretching the myocardium and improving contractility. However, when improved contractility as a compensation mechanism comes to an end, the left ventricle gradually dilates (remodelling) and its compliance increases (Fig. 1). Subsequently LV eccentric hypertrophy develops and causes diastolic as well as systolic LV dysfunction [1, 6]. In addition, according to the LaPlace law, wall stress is proportional to pressure and chamber size and inversely proportional to wall thickness.

$$\text{LV wall stress}=\frac{\text{LV pressure}\times \text{LV radius}}{2\times \text{LV wall thickness}}$$

**Fig. 2:**
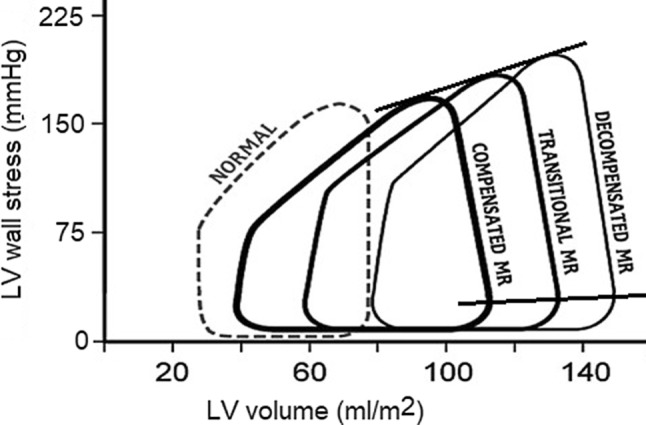
Left ventricular (*LV*) wall stress-volume loops in the three stages of chronic mitral regurgitation (*MR*). As the ventricle adapts to the chronic haemodynamic burden, a progressive increase in LV holosystolic as well as end-diastolic wall stress occurs. LV ejection fraction progressively declines from 65% in compensated MR, to 55% during the transitional stage, and finally to 45% (or lower) in decompensated MR. (Adapted from Gaasch and Meyer [1])

Thus, increased LVEDV and LV end-diastolic pressure (LVEDP) will result in an increased LV end-diastolic wall stress [1]. Also an increase in LV holosystolic wall stress (LVHSWS), representing afterload during the whole systole, is observed due to increased LV end-systolic volume (LVESV) (Fig. 2). Since myocardial blood perfusion depends on wall stress, increased LV wall stress may cause subendocardial ischaemia and subsequently myocardial fibrosis [1, 6–9].

Not only the left ventricle, but also the left atrium shows enlargement due to volume overload, which reflects the severity and duration of the MR. There is a close relationship between LA diameter and mortality, with a more than 3‑fold increase in mortality when the LA diameter ≥55 mm [10]. In contrast to the LV dimensions, the LA dimension is affected to a lesser degree by acute loading changes and therefore is a good marker for the average LA pressure for a longer time period.

## Lessons from MV surgery

In 1984, Zile et al. investigated the effect of several preoperative LV echocardiographic parameters on clinical symptoms after MV replacement in 20 patients with chronic MR [3]. They concluded that patients with preoperative LV end-diastolic dimension of more than 4.0 cm/m^2^, LVESD of more than 2.6 cm/m^2^ and LV end-systolic wall stress of more than 195 mm Hg (260 kdyn/cm^2^) remained symptomatic postoperatively, despite continued medical therapy. These observations led to the understanding that earlier intervention could lead to better outcome in these patients. More than 20 years later, Gaasch and Meyer proposed the use of three stages of chronic MR. Stage 1 consisted of a chronic compensated state with LV enlargement, eccentric hypertrophy and normal systolic function. Subsequently, there was a transitional phase (stage 2) with mild LV dysfunction that is reversible after surgical correction of the regurgitant lesion. Finally, stage 3 was a decompensated MR with progressive and irreversible structural and functional changes in the ventricle [1]. When analysing the LV wall stress-volume loops of these three stages, it is apparent that the LVEDV, LVESV and LVHSWS increase with every stage (Fig. 2). Moreover, the LV ejection fraction (LVEF) declines from 65% to 45% or less. The authors concluded with three recommendations. First, corrective surgery is indicated if the MR is severe. Second, the symptoms should be assessed and evaluated. In the absence of cardiac symptoms, many physicians hesitate to recommend surgery, unless clear and reliable LV dysfunction exists, or some other factor (e.g. the development of atrial fibrillation) must be considered. In contrast, surgery is indicated in symptomatic patients regardless of whether LV function is normal or abnormal (except for those with the most severe LV dysfunction). Third, the functional state of the left ventricle must be evaluated. Corrective surgery should be considered in an asymptomatic patient when the LVEF enters the transitional stage (LVEF 50–60%) and is strongly recommended before the LVEF is less than 50%. It should be noted that these recommendations are based on data from studies performed in the 1980s.

## Transcatheter treatment of MR

In 1998, Maisano et al. published an operative technique by means of which they approximated the mitral leaflets, creating a double-orifice MV, in order to diminish MR [11]. The technique was simple, easily reproducible and effective. Based on this edge-to-edge surgical technique, the MitraClip was developed and introduced in 2014. This percutaneous device can clip the edges of the MV leaflets in the same manner as the surgical technique proposed earlier. This method offered new opportunities for high-risk patients.

A decade ago, Siegel et al. studied the acute haemodynamic effects of MitraClip implantation by right heart catheterisation and echocardiography under general anaesthesia [12]. Significant improvements were found in cardiac output (5.0 l/min ± 2.0 to 5.7 l/min ± 1.9; *p* = 0.003), and better LV unloading manifested by a decrease in LVEDP (11.4 mm Hg ± 9.0 to 8.8 mm Hg ± 5.8; *p* = 0.016) and subsequently significantly lower systemic vascular resistance (1226 dyn · s/cm^−5^ ± 481 to 1004 dyn ·s/cm^−5^ ± 442; *p* < 0.001). Moreover, none of the patients had developed an acute postprocedural low cardiac output state, which occurred occasionally after surgical MV repair.

More recently, two important studies (MITRA-FR and COAPT studies) investigating transcatheter MV repair found conflicting results [13, 14]. The MITRA-FR study showed no difference between the intervention and the control group for the primary composite endpoint (mortality or heart failure hospitalisation), while the authors reporting on the COAPT trial concluded that the MitraClip procedure reduces the rates of hospitalisation for heart failure and all-cause mortality within 2 years of follow-up compared to medical therapy alone. The number needed to treat to prevent one hospitalisation for heart failure within 24 months was 3.1 patients. Apart from the number of patients in the two trials (304 vs 614), the baseline echocardiographic characteristics were also different. COAPT enrolled a subset of patients who had more severe MR and less advanced LV disease compared to MITRA-FR patients.

Furthermore, a more aggressive strategy for correction of MR was applied in COAPT, as suggested by the larger number of clips implanted per patient in COAPT than in MITRA-FR. Moreover, the rate of sustained reduction of MR was higher in COAPT than in MITRA-FR. The lower sustained efficacy of the MitraClip procedure may also have contributed to the lack of benefit of the intervention in MITRA-FR. Both trials required that patients remained symptomatic (New York Heart Association (NYHA) class ≥ II) despite the use of guideline-directed medical therapy (GDMT) for chronic systolic heart failure. However, COAPT imposed more demanding criteria for the inclusion of patients, namely the use of maximal tolerated doses of GDMT, and treatment with cardiac resynchronisation therapy, defibrillators and revascularisation, if appropriate. Hence, in COAPT, medical treatment was optimised prior to randomisation and only a few major adjustments in treatment occurred during follow-up. On the other hand, in MITRA-FR medical therapy was not optimised in all patients at baseline and multiple adjustments in medical treatment were allowed in each arm during follow-up. This issue may also have decreased the ability to reveal a beneficial effect of the intervention in MITRA-FR.

In view of these findings, Pibarot et al. concluded that, to achieve a reduction in heart failure hospitalisation and mortality by the MitraClip procedure, patients had to meet the following three criteria [15]:

1. ≥moderate-to-severe (grade 3+) secondary MR defined as effective regurgitant orifice area ≥30 mm^2^ and/or regurgitant volume >45 ml.
2. LVEF between 20% and 50% and LVESD <70 mm.
3. Persistent heart failure symptoms (NYHA ≥ II) despite optimal (maximally) tolerated GDMT with cardiac resynchronisation and coronary revascularisation if appropriate.

## Conclusion

Based on all these historical data, it can be expected that the benefit of treatment depends on the degree of MV disease. It is hypothesised that selection of patients, based on the criteria above, could be further improved by also measuring haemodynamic parameters as performed in the past [12, 16]. From this point of view, the LaPlace formula is important in understanding the various states of MR that can alter oxygen demand, resulting in subendocardial ischaemia and, subsequently, dyspnoea. Further insight will come from future studies.
